# Production of nitrogen fixing *Azotobacter* (SR-4) and phosphorus solubilizing *Aspergillus niger* and their evaluation on *Lagenaria siceraria* and *Abelmoschus esculentus*

**DOI:** 10.1016/j.btre.2019.e00323

**Published:** 2019-03-19

**Authors:** Misbahud Din, Rubina Nelofer, Muhammad Salman, Abdullah, Faisal Hayat Khan, Asad Khan, Munib Ahmad, Fazal Jalil, Jalal Ud Din, Mudassir Khan

**Affiliations:** a Department of Biotechnology, Abdul Wali Khan University, Mardan, Khyber Pakhtunkhwa, Pakistan; b Food and Biotechnology Research Centre, Pakistan Council of Scientific and Industrial Research (PCSIR), Laboratories Complex, Ferozpur Road, Lahore, 54600, Pakistan; c Department of Microbiology and Biotechnology, Abasyn University Peshawar, Pakistan; d Department of Healthcare Biotechnology, Atta-Ur-Rahman School of Applied Biosciences (ASAB), National University of Science and Technology (NUST), Islamabad, Pakistan; e Department of Biotechnology, Quaid-i-Azam University, Islamabad 44000, Pakistan

**Keywords:** Biofertilizers, *Azotobacter*, *A. niger*, Bottle gourd and okra

## Abstract

**Aims:**

Current study was aimed to produce nitrogen fixing *Azotobacter* sp. (SR-4) and phosphorus solubilizers *Aspergillus niger* (*A. niger*) and to evaluate their efficiency as biofertilizers for agricultural practices.

**Methods:**

Two biofertilizer including nitrogen fixing and phosphorus solubilizing were grown. The nitrogen fixing efficiency of *Azotobacter* (SR-4) was determined by Kjeldahl method. Similarly, Vanadomoybdate method was used to measure the soluble phosphorus while Heinonen method was used to analyze concentration of phytase and phosphatase in the cultures. Furthermore, both biofertilizers were tested in a field trail on *Lagenaria siceraria* (bottle gourd) and *Abelmoschus esculentus* (okra).

**Results:**

The *Azotobacter* (SR-4) strain was found efficient nitrogen fixer as 35.08 mg of nitrogen per gram of carbon was produced after 72 h of fermentation. Similarly, *A. niger* strain excrete extracellular phosphate solubilizing enzymes such as phytase (133UI in 48 h of fermentation) and phosphatase (170UI in 48 h of fermentation) which can solubilize the rock phosphate and make it available to plants. In field trials on selected plants (*L. siceraria* and *A. esculentus*), both biofertilizers showed significant increase in plant height, leaf length/width, fruit size and number of fruits per plant when compared with controls/untreated plants. Furthermore, plants co-inoculated with both the N fixing *Azotobacter* and phosphorus solubilizing *A. niger* have enhanced performance than those treated with each biofertilizer alone.

**Conclusion:**

The inoculation of seeds with *A. niger* and *Azotobacter* may replace costly and environment toxic chemical fertilizers with environment friendly and cost effective biofertilizers.

## Highlights

• Drawbacks of chemical fertilizers have attracted the attention of farmers toward biofertilizers.
• Nitrogen fixer *Azotobacter* SR-4 and phosphorus solubilizer *Asphergillus Niger* were produced and observed to be efficient biofertilzers.
• A significant increase in yield parameters were observed when these biofertilizers were evaluated on Bottle gourd and okra.
• In conclusion, biofertilizers or microbial inoculants could be used as an alternate source, which are both cost-effective as well as eco-friendly.

## 1 Background

Nitrogen (N) and phosphorus (P) are well-known fundamental nutrients needed by plant for their growth and development. To achieve high yield, farming practices require chemical fertilizers that are very costly and may also create environmental problems. Owed to environmental alarm and fear for consumer health, the use of chemical fertilizers in agriculture is presently under debate. Consequently, a specific group of fertilizers were discovered that are known as biofertilizers or bioinoculants and are consisted of microorganisms with plant growth-promoting abilities. Some of these microbial strains are capable of phosphorus solubilizing, nitrogen fixing from air and some produce cellulytic enzymes. Biofertilizers are applied in several ways to soil, to enhance the nutrient availability to the plants. One way is their direct application in soil, other way is seed treatment or application with composite. In either way the biofertilizers are used, they increase the numbers of beneficial microorganisms in the soil to enhance the nutrients availability for the plants.

A number of free-living nitrogen fixing bacteria have been reported as biofertilizers previously [[1], [2], [3]]. These bacteria greatly influence the plant growth when used as seed inoculants [4]. Some of them may affect the plants growth directly through synthesis of growth hormones, fixing nitrogen and solubilizing rock phosphates, when used as biofertilizers [[5], [6], [7]]. On the other hand, plant growth is also stimulated by phosphate-solubilizing microbes that enhance the available phosphorus and increase the uptake rate of nitrogen, potassium (K), and iron (Fe) [8]. These phosphate solubilizing microbes convert the insoluble phosphates into soluble form using different processes such as exchange reaction, acidification and chelation [9]. It has been reported in an earlier study that plants co-inoculated with biofertilizers have a significance increase in root and shoot biomass, nitrogenase activity and nitrogen fixation [[10], [11], [12], [13], [14]]. Moreover, combined inoculation of P-solubilizing and N-fixing biofertilizers were more effective compared to single inoculation due to the availability of more balanced nutrition for plants [15].

Therefore, in the present study, a bacterial strain *Azotobacter* (SR-4) and a fungal strain *A. niger* were used for nitrogen fixation and phosphorus-solubilization, respectively.

## 2 Methodology

### 2.1 Collection of cultures

The bacterial culture *Azotobacter* (SR-4) and fungal culture *A. niger* was obtained from the Food and Biotechnology Research Center (FBRC) of PCSIR, Labs. Complex, Lahore, Pakistan. This bacterial strain was previously isolated and identified at the same institute [16].

### 2.2 Inoculum preparation for nitrogen fixer

Stock culture of *Azotobacter* (SR-4) was maintained on nutrient agar slants and glycerol cultures in nutrient broth and was stored at −80℃. Inoculum was developed in Erlenmeyer flasks using nitrogen free (N-free) medium (2 g sucrose, 0.06 g K_2_HPO_4_, 0.016 g KH_2_PO_4_, 0.02 g NaCl, 0.02 g MgSO_4_, 0.05 g yeast extract, 0.01 g K_2_SO_4_, pH 7) and was incubated on a rotary shaker (360 rpm) at 30 °C for 24 h.

### 2.3 Inoculum preparation for phosphorus-solubilizer

*A. niger* was cultured in potato dextrose agar (PDA) slants for 3–4 days at 30 °C and fully-grown slants were stored at 4 °C for future use. 25 mL Pikovskayas agar (PVK) medium with following composition (g/L) [glucose, 10; yeast extract, 0.5; (NH_4_)_2_SO_4_, 0.5; MgSO_4_.7H_2_O, 0.1; KCl, 0.2; NaCl, 0.2; FeSO_4_.7H_2_O, 0.002; MnSO_4_.7H_2_O, 0.002; Ca_3_ (PO_4_)_5_, 5 (pH 7)] was taken in 250 mL Erlenmeyer flask and fungal culture was transferred to flask under aseptic conditions in LFH. The flasks were incubated for 7 days on a rotary shaker (200 rpm) at 30℃. All the ingredients of media were obtained from FBRC department of PCSIR Lahore, Pakistan. The efficiency of the culture was calculated for phosphorus solubilization in the inoculums medium.

### 2.4 Production of *Azotobacter* in small bioreactor

For fermentation, 1 L N-free media (2 g sucrose, 0.06 g K_2_HPO_4_, 0.016 g KH_2_PO_4_, 0.02 g NaCl, 0.02 g MgSO_4_, 0.05 g yeast extract, 0.01 g K_2_SO_4_, pH 7) was prepared in 1 L bioreactor. 50 ml *Azotobacter* (SR-4) inoculum, maintained in N-free media, was transferred to bioreactor. Samples were collected from bioreactor daily up to 6 days and were then analyzed for nitrogen.

### 2.5 Solid-State Fermentation for *A. niger*

For solid-state fermentation, 1 kg wheat bran was moistened with 500 ml water and was sterilized in autoclave at 121 °C for 15 min. After cooling to room temperature, the culture of *A. niger* was inoculated as inoculum and then incubated for 8 days at 30 °C in a static inclined position in a steel tray covered with aluminum foil having small pores.

### 2.6 Evaluation of biofertilizers

The effect of these biofertilizers on plant’s height, length and width of leaf and the number of fruits per plant was tested in *L. siceraria* and *A. esculentus*. For this purpose, three treatments were applied; In 1^st^ treatment, 500 g plant seeds were inoculated with 250 g phosphorus solubilizing biofertilizer, in the 2^nd^ treatment seeds were treated with 250 mL nitrogen fixing biofertilizer and in the 3^rd^ treatment seeds were co-inoculated with both phosphorus (250 g) and nitrogen biofertilizers (250 mL) (Table 1). The bio-fertilizers were used to make slurry with water (phosphorus solubilizing) and soil (nitrogen fixing), and the slurry coated seeds were then air dried and were sown in soil. However, same quantity of untreated seeds was used as control.

**Table 1:** Treatments scheme for 500 g seed of *Lagenaria siceraria* and *Abelmoschus esculentus* plants.

Sr. No.	Bottle guard seeds treatments		Okra seeds treatments	
	Amount of Nitrogen fixing biofertlizer	Amount of phosphorus solubilizing fertilizer	Amount of Nitrogen fixing biofertlizer	Amount of phosphorus solubilizing fertilizer
**T1**	250 mL	--	250 mL	--
**T2**	--	250 mg	--	250 mg
**T3**	250 mL	250 mg	250 mL	250 mg
Control	--	--	--	--

### 2.7 Analytical methods

#### 2.7.1 Efficiency of *Azotobacter* as nitrogen fixer

The nitrogen fixing efficiency of *Azotobacter* (SR-4) is equal to the mg of nitrogen produced per gram of carbon utilized. The nitrogen efficiency of *Azotobacter* (SR-4) was determined by the Kjeldahl method [17]. The samples were centrifuged at 4000 rpm and 4 °C for 10 min. Then 2 ml of supernatant was mixed with 10 ml K_2_Cr_2_O_7_ solution and 20 ml of H_2_SO_4_ and was heated for 1 min. In the following step, 200 ml of H_2_O was added again with 4–5 drops of ferroin indicator. Titration of the above solution was conducted against 0.5 N FeSO_4_ solution and total carbon was measured from the total volume of FeSO_4_ solution used.

#### 2.7.2 Efficiency of *A. niger* as phosphorus solubilizer

Similarly, in determination of phosphorus solubilizing activity, the samples were centrifuged at 4000 rpm and 4 ℃ for 10 min and supernatant was collected. Vanadomolybdate reagent was prepared by mixing ammonium molybdate 5% (W/V), ammonium vanadate 0.25% (W/V) and diluted nitric acid with water in 1:3 ratios and was used to measure the soluble phosphorus [18]. However, Heinonen method was used to analyze phytase and phosphatase [19]. 1 mL of supernatant was incubated with same quantity of phytic acid and tricalcium phosphate (substrate for phytase and phosphatase) in 200 mM glycine buffer (pH 5) at 35 ℃ for 1 h. After incubation, 1 mL citric acid (1 M) was added to stop enzyme activity. In the last step, 4 mL of reagent mixture containing 2.5% (W/V) solution of ammonium molybdate, 5 N H_2_SO_4_ and acetone in 1:1:2 ratio was added, vortexed and the optical density was observed at 400 nm. 1 IU for phosphatase and phytase was equal to 1 μM of phosphorus released/mL/min.

#### 2.7.3 Statistical analysis

The findings were statistically compared through LSD test using SPSS V16. The significant difference was represented with different letters and non-significant difference was represented with similar letters with yield values.

## 3 Results

### 3.1 Nitrogen fixing efficiency of *Azotobacter*

Carbon utilization and nitrogen fixation was determined at different intervals of fermentation. For this purpose, samples (n = 6) were collected after every 12 h for 3 days of fermentation. After 12 h of incubation, carbon utilization was 0.34 g/100 mL and was increased to 0.61 g/100 mL by 72 h. Similarly, N fixation capacity was increased from 8.0 mg/100 mL to 21.40 mg/100 mL by 72 h of incubation. Consequently, with increase in cell mass the carbon contents of medium decreases lead to increased utilization of carbon. The efficiency of *Azotobacter* (SR-4) (mg of nitrogen produced per gram of carbon utilized) was 23.52 N mg/gC after 12 h and increased to 35.08 N mg/gC with increase in fermentation time to 72 h (Table 2).

**Table 2:** Nitrogen fixing efficiency of Azotobacter (SR-4).

Sr. No.	Fermentation Time	Measured nitrogen (mg/100 mL)	Carbon utilized (g/100 mL)	Efficiency (mg of N/g of carbon)
1	12 h	08.00	0.34	23.52
2	24 h	10.60	0.42	25.23
3	36 h	14.00	0.49	28.57
4	48 h	16.20	0.54	30.00
5	60 h	18.80	0.58	32.41
6	72 h	21.40	0.61	35.08

### 3.2 Phosphorus solubilizing efficiency of *A. niger*

Phosphorus solubilizing efficiency was measured in solid state fermentation at different intervals of fermentation. Enzymatic activity was increased from 0-170IU for phosphatase and 0-133IU for phytase during 0–48 h of incubation. However, decline in concertation of both phosphatase and phytase was observed after 48 h. Similarly, maximum soluble phosphorus of 835 ppm was observed after 48 h of incubation which support the increased production of phosphate degrading enzymes by *A. niger* (Table 3)

**Table 3:** Phosphorus solubilizing efficiency of *A. niger.*

Sr. No.	Fermentation time (h)	Phosphorus (ppm)	Phosphatase (IU)	Phytase (IU)
1	0	0	0	0
2	4	165	0	0
3	8	275	18	0
4	12	360	42	19
5	16	400	84	58
6	20	475	117	112
7	24	710	145	125
8	36	785	154	131
9	48	835	170	133
10	60	775	152	125
11	72	690	136	120

### 3.3 Biofertilizers effect on *Lagenaria siceraria* and *Abelmoschus esculentus*

The field trials of biofertilizers on selected plants (*L. siceraria* and *A. esculentus*) showed significant increase in plant height, leaf length/width, fruit size and number of fruits per plant when compared with controls/untreated plants. Furthermore, plants co-inoculated with both the N fixing *Azotobacter* and phosphorus solubilizing *A. niger* have enhanced performance than those treated with each biofertilizer alone (Table 4, Table 5).

**Table 4:** Effects of biofertilizers on *Lagenaria siceraria*.

Parameters	Treatment 1 (P)	Treatment 2 (N)	Treatment 3 (P + N)	Control
Number of plants	(19 plants)	(18 plants)	(33 plants)	(9 plants)
Plant height	142.8 ± 18.30^b^	141.2 ± 15.02^b^	160 ± 14.87^c^	119.77 ± 13.58^a^
Leaf length/ width	4.21 ± 0.47^b^	4 ± 0.43^b^	4.73 ± 0.42^b^	3.24 ± 0.2^a^
Fruit length / width	4.17 ± 0.39^b^	4.11 ± 0.28^b^	4.8 ± 0.2^c^	3.3 ± 0.14^a^
No. of fruits/plant	11 ± 2^bc^	9 ± 3^b^	15 ± 2^b^	7 ± 2^ab^

The values with different letters are significantly different.

**Table 5:** Effects of biofertilizers on *Abelmoschus esculentus*.

Parameters	Treatment 1 (P)	Treatment 2 (N)	Treatment 3 (P + N)	Control
Number of plants	(66 plants)	(63 plants)	(108 plants)	(36 plants)
Plant height	35.7 ± 3.2^b^	35.76 ± 3.21^b^	43 ± 2.82^c^	26.2 ± 3.72^a^
Leaf length/ width	5.8 ± 0.51^bc^	5.3 ± 0.49^b^	6.4 ± 0.32^c^	3.84 ± 0.2^a^
Fruit length/ width	6.2 ± 0.39^b^	6.2 ± 0.39^b^	6.94 ± 0.2^c^	4.9 ± 0.14^a^
No. of fruits/plant	26 ± 3^ab^	29 ± 4^bc^	35 ± 3^c^	21 ± 2^a^

The values with different letters are significantly different.

## 4 Discussion

The bio-fertilizers due their environment friendly nature as compared to the chemical fertilizers is a method of choice for the modern agriculture. Therefore, the present research work was aimed to produce and evaluate *Azotobacter* (SR-4) as a nitrogen fixer and *A. niger* as phosphorus solubilizer, that are important constituents needed by the plants. These strains were obtained from the Food and Biotechnology Research Center (FBRC) of PCSIR, Labs. Complex, Lahore, Pakistan. Their inoculums were developed and maintained and were grown in large scale. Then their efficiency as biofertilizers were evaluated and tested in field trails on selected plants in three different treatment schemes.

*Azotobacter* species have been known to releases variety of growth-promoting substances in addition to nitrogen like indole acetic acid, vitamins B and gibberellins [20,21]. Similarly, *Azotobacter* species excrete ammonia in the rhizosphere hence helps in plant improvement [22]. In the present study, the N-fixation capacity of *Azotobacter* (SR-4) was measured by analyzing the concertation of nitrogen in the medium using Kjildhal method [17]. It has been reported that the excess of carbon compound and shortage of combined nitrogen in the media greatly affect the activity of nitrogen fixing microorganisms [23]. Increase in *Azotobacter* (SR-4) activity was observed with increase incubation time. This increase in activity is due to increase in cell mass and hence the increased nitrogenase enzymes production by *Azotobacter* that fix nitrogen (Table 2). Our findings of increase in efficiency of Azotobacte with increase in incubation time is consistent with other studies, where similar trend in behavior has been reported [24]. Furthermore, we observed higher nitrogen fixing efficiency as compared to other published reports [25,26]. Variation in efficiency of *Azotobacter* may be due to difference in strains being used in different studies.

Sometimes, the efficiency might be different due to amount of dissolved oxygen that affect the carbon consumption rate as well as nitrogen fixation. Although, it has been shown that *Azotobacter* sp. is usually considered as nitrogen fixer, however, addition of small quantity of nitrogen in the medium reduce the fermentation time due to short lag phase and generation time [25]. This assumption was tested by increasing the incubation time and hence maximum nitrogen fixation was observed in the current study. Due to this N-faxing capability of *Azotobacter*, extracellular proteins and ammonia are secreted in nitrogen free medium that are accessible to the plants. Similarly, *Azotobacter* (SR-4) was found to be efficient nitrogen fixer able to fix 23.52–35.08 mg N/g of carbon oxidized which correspondent to 8–21.40 mg N/g sucrose consumed within 72 h (Table 2). A similar trend of increase in cell count with increase in incubation time has been previously observed which reaches to its peak after two weeks [26]. Although, an aerobic condition is required for growth of *Azotobacter*, while low oxygen tension or anaerobic conditions are optimum for enzymatic activity of a system, such as nitrogenase responsible for nitrogen fixation. Similarly, compare to liquid media, soil provides more suitable condition for *Azotobacter* growth and subsequently nitrogen fixation by providing superior equilibrium between anaerobiosis and aerobiosis.

We observed an efficient phosphorus solubilizing activity in *A. niger* which agrees to previous reports [[27], [28], [29]]. A significant increase in the concertation of phytase, phosphatase and soluble phosphorus was also found after 48 h of fermentation along with decrease in soluble phosphorus concentration. This decrease may be due to utilization of phosphorous by fungus mycelia. Our findings are consistent to an earlier study where *Aspergillus* has shown maximum growth after incubation of 50 h under favorable conditions [30].

The increase in concentration of enzymes observed in our study is higher than that reported in literature [31]. Although, fungi produce these enzymes to solubilize phosphate and phytic acid for its own growth, consequently, a considerable amount of phosphorus become available to plants as well. These N fixing and phosphorus solubilizing biofertilizer were tested in field trails on selected plants in 3 different treatments (Table 4, Table 5). Previous studies have reported that co-inoculation of strains has enhanced root growth, shoot biomass, N% as well as total plant nitrogen in many crops [[10], [11], [12]]. Similarly, co-inoculations have considerably increased the yields as compared to single inoculation in soybean, pea, chickpea, groundnut, mungbean and in other crops [[31], [32], [33], [34], [35], [36], [37]]. There are several reports available on the inevitability and applicability of bacterial fertilizers and phosphate dissolving and N_2_-fixing bacteria [24,[38], [39], [40], [41], [42]]. Azotobactor when applied to green gram and rice have shown a significant improvement in seed germination and root nodules [42]. Furthermore, *Aspergillus* sp. has been considered as plant growth promoting fungi by solubilizing phosphorus and can travel long distance as compared to bacteria [39,41]. Interestingly, the co-inoculation of phosphorus solubilizing fungi and nitrogen fixing bacteria were found effective in overcoming drought stress in legume plants as well [40].

Considering these fact and findings it can be said that the effects can be highly variable in practical agriculture. However, it further signifies that combined inoculation of seeds with *A. niger* and *Azotobacter* may replace costly and environment toxic fertilizers with environment friendly biofertilizers [43].

## 5 Conclusion

It is concluded that biofertilizers, including the nitrogen fixing and phosphorus solubilizing biofertilizer, can be produced cost effectively and can be used for the sustainable and environmental friendly yield enhancement of vegetables. Higher yield than the control showed that the produced biofertilizers have no adverse effect on plant growth of bottle guard and okra. Therefore, the used microbial strains have been proved potential for the routine agriculture practices with additional benefits either separately or in combination or may be supplemented to the chemical fertilizers to reduce their impact on environment.

## Conflict of interest

There is no conflict of interest in the research work.
